# A comparative analysis of clinical outcomes between conversion to mTOR inhibitor and calcineurin inhibitor reduction in managing BK viremia among kidney transplant patients

**DOI:** 10.1038/s41598-024-60695-2

**Published:** 2024-06-04

**Authors:** Ara Cho, Sunghae Park, Ahram Han, Jongwon Ha, Jae Berm Park, Kyo Won Lee, Sangil Min

**Affiliations:** 1 Department of Surgery, Seoul National University College of Medicine, Seoul, South Korea; 2 Department of Surgery, Samsung Medical Center, Seoul, South Korea

**Keywords:** Virology, Viral infection

## Abstract

BK virus-associated nephropathy (BKVAN) exerts a substantial impact on allograft survival, however, the absence of robust clinical evidence regarding treatment protocols adds to the complexity of managing this condition. This study aimed to compare the two treatment approaches. The study population consisted of patients who underwent kidney transplantation between January 2016 and June 2020 at two tertiary hospitals in Korea. Patients diagnosed with BK viremia were evaluated based on their initial viral load and the treatment methods. The ‘Reduction group’ involved dose reduction of tacrolimus while the ‘Conversion group’ included tacrolimus discontinuation and conversion to sirolimus. A total of 175 patients with an initial viral load (iVL) ≥ 3 on the log10 scale were evaluated within two iVL intervals (3–4 and 4–5). In the iVL 4–5 interval, the Reduction group showed potential effectiveness in terms of viral clearance without statistically significant differences. However, within the iVL 3–4 interval, the Reduction group demonstrated superior viral clearance and a lower incidence of biopsy-proven acute rejection (BPAR) than the Conversion group. The renal function over 12 months after BKV diagnosis showed no statistically significant difference. Reducing tacrolimus compared to converting to mTORi would be a more appropriate treatment approach for BK viral clearance in kidney transplantation. Further research is warranted in a large cohort of patients.

## Introduction

The BKV is an opportunistic pathogen capable of reactivation in kidney transplant recipients, leading to the development of tubule-interstitial nephropathy^1,2^. BK virus-associated nephropathy (BKVAN) frequently occurs within the first year following KT and is known to be caused by high levels of immunosuppression leading to the reactivation of latent BK polyomavirus in the recipient and subsequent enhancement of BKV infection in the allograft^3,4^. This condition can lead to chronic allograft failure and even graft loss in up to 50% of cases in the pre-screening era. BKVAN is observed in approximately 5% of renal transplant patients, and effective management of this infection could exert a substantial influence on allograft survival^5^.

Balancing rejection and infection in KT is a complex task that presents several challenges^6^. One of the main difficulties is using immunosuppressive therapy, which is essential to prevent rejection but can also increase the risk of infection, including BK viremia. Moreover, limited treatment options are available for BK viremia, and the optimal management strategy remains unclear. Further research is needed to better understand the pathogenesis of BKVAN and develop more effective prevention and treatment strategies.

Despite its clinical significance, there is a discrepancy in treatment between hospitals and clinicians because of insufficient research on this matter. To address this gap, we conducted a retrospective cohort study to compare the outcomes of different treatment methods for BKV-associated diseases. By analyzing medical records, we aimed to evaluate the efficacy and safety of various treatment approaches, such as the reduction of the calcineurin inhibitor (CNI) and antimetabolites, and conversion to the mammalian target of rapamycin inhibitor (mTORi). Additionally, we explored potential factors that may influence treatment outcomes, such as patient demographics, serum BK viral loads, and the timing of intervention.

## Results

### Patient demographics and baseline clinical characteristics

**Figure 1:**
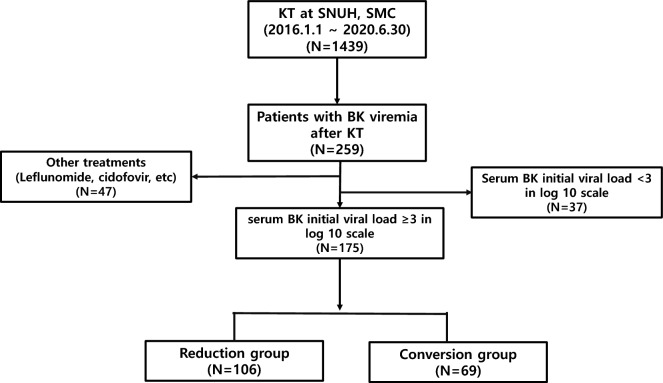
Patient selection. SNUH, Seoul National University Hospital; SMC, Samsung Medical Center; KT, Kidney transplantation.

During the study period, a total of 1439 patients underwent KT at the two institutions. Among them, 259 patients (18.0%) were identified as having BK viremia during post-transplant screening. A total of 259 patients were initially enrolled in the study, with 47 patients excluded due to receiving different treatment strategies. Thirty-seven patients were further excluded because their BK viral load was less than 3 on the log10 scale. Consequently, the evaluation was conducted on the final cohort of 175 patients who had an initial viral load of ≥ 3 on the log10 scale. Within this group, 106 patients were classified into the Reduction group, and 69 patients into the Conversion group (Fig. 1).

In the entire study population, males constituted 63.4% of the cohort, with a median age of 54.0 (range: 44.0–61.0). The leading etiology of end-stage renal disease was diabetes mellitus (DM), accounting for 31.4% of cases, excluding other causes. ABO incompatibility, preformed anti-HLA donor-specific antibody (DSA) positivity, and positive crossmatches (XM) were observed in 15.4%, 15.8%, and 6.9%, respectively. Re-transplantation was performed in 10 cases. Induction therapy with basiliximab was administered in 37.1% of cases, while Anti Thymocyte Globulin (ATG) was used in 62.3% of cases. Rituximab treatment was undertaken in 28.0% of cases. Demographics and baseline clinical characteristics are summarized in Table 1.

**Table 1:** Demographics and baseline clinical characteristics.

	Total (N = 175)	Reduction group (N = 106)	Conversion group (N = 69)	*p-value*
Male, n (%)	111 (63.4)	66 (62.3)	45 (65.2)	0.692
Age, median (IQR)	54 (44.0–61.0)	53.5 (43.0–60.0)	54.0 (44.0–62.0)	0.732
BMI, median (IQR)	22.5 (20.3–25.0)	22.6 (20.4–25.6)	22.1 (20.2–24.0)	0.274
Cause of ESRD				0.978
DM, n (%)	55 (31.4)	34 (32.1)	21 (30.4)	
HTN, n (%)	17 (9.7)	9 (8.5)	8 (11.6)	
PKD, n (%)	5 (2.9)	4 (3.8)	1 (1.4)	
IgAN, n (%)	32 (18.3)	19 (17.9)	13 (18.8)	
Others, n (%)	66 (37.7)	40 (37.7)	26 (37.7)	
ABO incompatibility, n (%)	27 (15.4)	20 (18.9)	7 (10.1)	0.118
Preformed DSA+, n (%)ns	27 (15.8)	15 (14.4)	12 (17.9)	0.542
XM+, n (%)	12 (6.9)	7 (6.6)	5 (7.2)	1.000
Donor type				< 0.001
LDKT, n (%)	100 (57.1)	74 (69.8)	26 (37.7)	
DDKT, n (%)	75 (42.9)	32 (30.2)	43(62.3)	
Re-transplantation, n (%)	10 (5.7)	5 (4.7)	5 (4.7)	0.518
Induction agent				
Basiliximab usage, n (%)	65 (37.1)	51 (48.1)	14 (20.3)	< 0.001
ATG usage, n (%)	109 (62.3)	54 (50.9)	55 (79.7)	< 0.001
Rituximab treatment, n (%)	49 (28.0)	30 (28.3)	19 (27.5)	0.912
BPAR before BK diagnosis, n (%)	35 (20.0)	25 (23.6)	10 (14.5)	0.142
Pulse treatment before BK diagnosis, n (%)	30 (17.1)	21 (19.8)	9 (13.0)	0.246
Timing of BK diagnosis (postop month), median (IQR)	3.0 (2.0–6.0)	3.0 (2.0–7.0)	2.0 (1.0–5.0)	< 0.001
Initial viral load (log_10_ copies/mL), median (IQR)	3.6 (3.2–4.2)	3.4 (3.2–3.8)	4.1 (3.5–4.9)	< 0.001
Follow-up months, median (IQR)	45.0 (34.0–58.0)	43.0 (31.8–58.3)	47.0 (37.0–58.0)	0.163
FK trough level (ng/ml) at start of treatment	8.2 (6.6–9.7)	8.1 (6.4–9.8)	8.2 (6.8–9.9)	0.733

BMI, body mass index; ESRD, end stage renal disease; DM, diabetes mellitus; HTN, hypertension; PKD, polycystic kidney disease; IgAN, immunoglobulin A nephropathy; DSA, donor specific antibody; XM, crossmatch; LDKT, living donor kidney transplantation; DDKT, deceased donor kidney transplantation; ATG, anti thymocyte globulin; BPAR, biopsy proven acute rejection; FK, tacrolimus; IQR, interquartile range.

A total of 35 patients (20.0%) were diagnosed with BPAR before the occurrence of BK viremia. Before the diagnosis of BK viremia, 17.1% of cases received steroid pulse treatments. The diagnosis of BK viremia occurred at a median of 3.0 months postoperatively, ranging from 2.0 to 6.0 months. The median initial viral load at the initiation of treatment was 3.6 copies/mL on the logarithmic scale, with a range of 3.2 to 4.2 copies/mL. Patients were followed up for a median duration of 44.0 months, ranging from 34.0 to 58.0 months.

Deceased donor kidney transplantation (DDKT) accounted for a relatively large proportion (62.3%) in the Conversion group while living donor kidney transplantation (LDKT) was more prevalent in the Reduction group (p < 0.001). ATG was used in 50.9% and 79.7% of cases in the Reduction and Conversion group, respectively. This indicated a higher usage of ATG in the Conversion group than in the Reduction group (p < 0.001). Patients in the Reduction group were diagnosed with BK viremia at a median of 3.0 (range 2.0–7.0) months postoperatively, while patients in the Conversion group received their BK viremia diagnosis at a median of 2.0 (range 1.0–5.0) months postoperatively (p < 0.001). When comparing the median initial viral load at the diagnosis of BK viremia, the Reduction group showed a value of 3.4 (range 3.2–3.8), and the Conversion group showed a value of 4.1 (range 3.5–4.9), with significant differences (p < 0.001). The median tacrolimus trough level at the start of the BK viremia treatment was 8.1 (range 6.4–9.8) in the Reduction group and 8.2 (range 6.8–9.9) in the Conversion group, without significant differences (p = 0.733).

### Clinical outcomes

Patients were stratified into two subgroups based on their initial viral load: those with an initial viral load ranging from 3 to 4 and those with an initial viral load ranging from 4 to 5 on the log10 scale. Subsequently, the clinical outcomes of the Reduction and the Conversion groups were compared within each of these two subgroups. The results are presented in Table 2.

**Table 2:** Clinical outcomes.

	Initial VL 3–4 in log 10 scale			Initial VL 4–5 in log 10 scale		
	Reduction group (N = 92)	Conversion group (N = 31)	*p-value*	Reduction group (N = 13)	Conversion group (N = 26)	*p-value*
Persistent viremia after 3 months of treatments, n (%)	30 (32.6)	12 (38.7)	0.536	7 (53.8)	17 (65.4)	0.485
BPAR after treatment, n (%)^a^	14/75 (18.7)	15/28 (53.6)	< 0.001	7/8 (87.5)	12/25 (48.0)	0.651
Graft loss, n (%)	3 (3.3)	1 (3.2)	1.000	0 (0.0)	0 (0.0)	
Patient death, n (%)	2 (2.2)	3 (9.7)	0.101	0 (0.0)	1 (3.8)	1.000
De novo DSA, n (%)^a^	10 (10.9)	4 (12.9)	0.750	1 (7.7)	3 (11.5)	1.000
Peak viral load (log_10_ copies/mL), median (IQR)	3.4 (3.2–3.8)	4.1 (3.7–4.9)	< 0.001	4.3 (4.2–4.7)	4.8 (4.4–4.9)	0.171
Velocity (log_10_ copies/mL/month), median (IQR)	3.0 (2.7–3.3)	2.8 (2.5–3.2)	0.139	3.8 (3.3–3.9)	3.9 (3.5–4.2)	0.315
Time duration for clearance, median (IQR)	2.0 (2.0–4.0)	3.0 (2.0–5.5)	0.035	3.5 (2.0–9.5)	4.5 (3.0–6.0)	0.515

VL, viral load; BPAR, biopsy proven acute rejection; DSA, donor specific antibody; IQR, interquartile range.

^a^Within 1 year after the diagnosis of BK viremia.

In the initial viral load (iVL) range of 3–4 on the log 10 scale, there were 92 patients in the Reduction group and 31 patients in the Conversion group. In these iVL patients, the Reduction group showed comparable viral clearance and a lower incidence of biopsy-proven acute rejection (BPAR) compared to the Conversion group. After 3 months of treatment, the proportion of patients with persistent viremia was 32.6% and 38.7% in the Reduction and Conversion group, respectively (p = 0.536). During the 1-year follow-up after diagnosis and treatment, BPAR occurred in 18.7% and 53.6% of patients, respectively (p < 0.001). Graft loss was observed in 3.3% and 3.2% of patients, respectively (p = 1.000). The patient death occurred in 2.2% and 9.7% of patients, respectively (p = 0.101). The de novo donor-specific anti-HLA antibodies (DSA) were detected in 10.9% and 12.9% during the 1-year follow-up, respectively (p = 0.750). The peak serum viral load was 3.4 (range 3.2–3.8) and 4.1 (range 3.7–4.9), respectively (p < 0.001). The time duration for clearance was significantly shorter in the Reduction group compared to the Conversion group (2.0 (range 2.0–4.0) vs 3.0 (range 2.0–5.5), p = 0.035). The occurrence of BK virus-associated nephropathy (BKVAN) within 1 year after treatment was also compared. Due to variations in biopsy protocols across hospitals, it was not possible to compare the proportions directly. However, in the Reduction group, BKVAN was confirmed in 3 out of 75 biopsies (4.0%), while in the Conversion group, it was observed in 10 out of 28 biopsies (35.7%).

In the initial viral load (iVL) range of 4–5 on the log 10 scale, there were 13 patients in the Reduction group and 26 patients in the Conversion group. After 3 months of treatment, the proportion of patients with persistent viremia was 53.8% and 65.4%, respectively (p = 0.485). During the 1-year follow-up after diagnosis and treatment, biopsy-proven acute rejection (BPAR) occurred in 87.5% and 48.0% of cases of the Reduction and Conversion group, respectively (p = 0.651). The graft loss was not observed in either group, while patient death occurred in 1 case (3.8%) in the Conversion group. The de novo donor-specific antibodies (DSA) were detected in 7.7% and 11.5% of cases during the 1-year follow-up, respectively (p = 1.000). The peak viral load was 4.3 (4.2–4.7) and 4.8 (4.4–4.9), respectively (p = 0.171). The time duration for clearance was 3.5 (2.0–9.5) and 4.5 (3.0–6.0), respectively without statistically significant differences (p = 0.515). The BK virus-associated nephropathy (BKVAN) was confirmed in 1 out of 8 biopsies (12.5%) in the Reduction group and 9 out of 25 biopsies (36.0%) in the Conversion group during the 1-year follow-up.

### Changes of eGFR after BKV diagnosis for 12 months of follow-up

**Figure 2:**
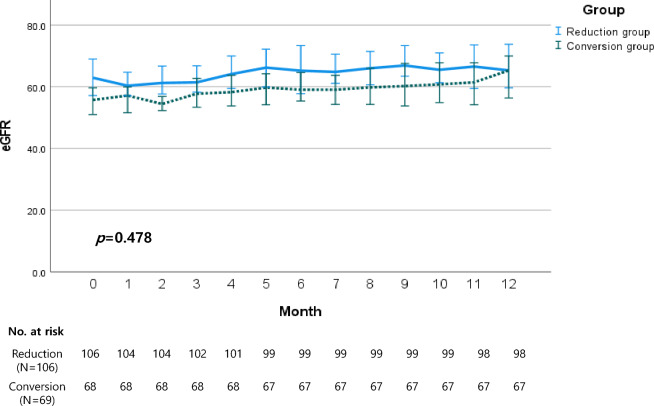
Interval changes of estimated glomerular filtration rate (eGFR) over 12 months following the diagnosis of BK viremia.

The changes in estimated glomerular filtration rate (eGFR) over 1-year following BK virus (BKV) diagnosis were tracked in both groups. At the time of BKV diagnosis, the median eGFR for the Reduction group was 62.9 (range 48.1–80.0), and for the Conversion group, it was 55.7 (range 45.1–71.9) without significant differences (p = 0.065). Six months later, the median eGFR was 65.2 (range 53.3–80.7) for the Reduction group and 59.0 (range 49.2–70.8) for the Conversion group, without significant differences (p = 0.059). After 1 year, the median eGFR was 65.3 (range 53.9–80.6) for the Reduction group and 65.3 (range 47.3–76.5) for the Conversion group, without significant differences (p = 0.174). The difference in median eGFR between the two groups did not reach statistical significance (p = 0.478) and was not aggravated over 1 year, as illustrated in Fig. 2.

### Risk factors of persistent viremia after 3 months of treatment and BPAR within 1 year after diagnosis

The baseline characteristics and factors related to BKV infection were analyzed and some factors were statistically significant in multivariable logistic analysis. The risk factors for persistent viremia after 3 months were identified as later BK viremia diagnosis (odds ratio [OR] 1.08, 95% confidence interval [CI] 1.01–1.16, p = 0.026) and higher peak viral load (OR 4.49, 95% CI 2.60–7.75, p < 0.001) as shown in Table 3.

**Table 3:** The risk factors of persistent viremia after 3 months of treatments.

	Univariable			Multivariable		
	OR	95% CI	*p-value*	OR	95% CI	*p-value*
Male	0.64	0.35–1.19	0.159			
Age	1.00	0.98–1.02	0.839			
BMI	0.95	0.88–1.03	0.239			
Cause of ESRD						
DM	(ref)					
HTN	1.13	0.37–3.43	0.825			
PKD	0.41	0.04–3.87	0.432			
IgAN	1.62	0.67–3.91	0.284			
Others	1.62	0.78–3.35	0.194			
ABO incompatibility	1.19	0.52–2.70	0.685			
Preformed DSA+	1.03	0.45–2.35	0.947			
XM+	0.88	0.27–2.89	0.834			
Donor type						
LDKT	0.43	0.43–1.43	0.430			
DDKT	(ref)					
Re-transplantation	1.26	0.35–4.52	0.723			
Basiliximab usage	1.35	0.73–2.50	0.341			
ATG usage	0.70	0.38–1.30	0.262			
Rituximab treatment	1.02	0.53–1.98	0.957			
Timing of BK diagnosis (postop month)	1.06	1.00–1.12	0.068	1.08	1.01–1.16	0.026
Initial viral load (log_10_ copies/mL)	2.98	1.80–4.93	< 0.001	0.68	0.26–1.79	0.434
Peak viral load (log_10_ copies/mL)	3.21	2.02–5.11	< 0.001	4.49	2.60–7.75	< 0.001
Velocity (log_10_ copies/mL/month)	1.04	0.66–1.66	0.862			
Treatment						
Reduction group	(ref)			(ref)		
Conversion group	2.47	1.33–4.60	0.004	1.02	0.43–2.47	0.958
FK trough level (ng/ml) at the start of treatment	0.91	0.81–1.01	0.072	0.89	0.79–1.00	0.051

BMI, body mass index; ESRD, end stage renal disease; DM, diabetes mellitus; HTN, hypertension; PKD, polycystic kidney disease; IgAN, immunoglobulin A nephropathy; DSA, donor specific antibody; XM, crossmatch; LDKT, living donor kidney transplantation; DDKT, deceased donor kidney transplantation; ATG, anti thymocyte globulin; BPAR, biopsy proven acute rejection; FK, tacrolimus; OR, odd ratio; CI, confidence interval; ref, reference.

In addition, the risk factors for BPAR within 1 year after diagnosis were identified. The risk factors were lower initial viral load (OR 0.08, 95% CI 0.01–0.50, p = 0.007), higher peak viral load (OR 3.86, 95% CI 1.35–11.07, p = 0.012), higher velocity (OR 10.43, 95% CI 2.25–48.33, p = 0.003), and the conversion of CNI to mTORi (OR 2.37, 95% CI 1.00–5.64, p = 0.051) as shown in Table 4.

**Table 4:** The risk factors of BPAR within 1 year.

	Univariable			Multivariable		
	OR	95% CI	*p-value*	OR	95% CI	*p-value*
Male	0.90	0.47–1.75	0.765			
Age	0.99	0.97–1.01	0.371			
BMI	1.02	0.94–1.11	0.632			
Cause of ESRD						
DM	(ref)					
HTN	1.99	0.65–6.03	0.226			
PKD	3.35	0.51–21.94	0.207			
IgAN	0.75	0.28–1.99	0.558			
Others	0.90	0.41–1.97	0.799			
ABO incompatibility	0.91	0.37–2.22	0.827			
Preformed DSA+	1.14	0.47–2.73	0.775			
XM+	0.71	0.19–2.74	0.621			
Donor type						
LDKT	0.50	0.26–0.96	0.036			
DDKT	(ref)					
Re-transplantation	0.93	0.23–3.75	0.920			
Basiliximab usage	0.53	0.26–1.05	0.069			
ATG usage	1.97	0.98–3.95	0.056			
BPAR before BK diagnosis	0.59	0.25–1.39	0.225			
Pulse treatment before BK diagnosis	0.38	0.14–1.05	0.063			
Rituximab treatment	1.23	0.61–2.48	0.562			
Timing of BK diagnosis (postop month)	0.88	0.81–0.97	0.009	0.91	0.83–1.01	0.068
Initial viral load (log_10_ copies/mL)	1.87	1.21–2.90	0.005	0.08	0.01–0.50	0.007
Peak viral load (log_10_ copies/mL)	2.16	1.43–3.27	< 0.001	3.86	1.35–11.07	0.012
Velocity (log_10_ copies/mL/month)	2.19	1.30–3.67	0.003	10.43	2.25–48.33	0.003
Treatment						
Reduction group	(ref)			(ref)		
Conversion group	3.93	2.01–7.70	< 0.001	2.37	1.00–5.64	0.051
FK trough level (ng/ml) at the start of treatment	0.93	0.83–1.05	0.241			
Persistent viremia after 3 month of treatments	1.31	0.69–2.48	0.416			
Time to clearance, per month	0.95	0.87–1.03	0.229			
De novo DSA	1.69	0.64–4.46	0.292			
BKVAN	1.75	0.79–3.90	0.168			

BMI, body mass index; ESRD, end stage renal disease; DM, diabetes mellitus; HTN, hypertension; PKD, polycystic kidney disease; IgAN, immunoglobulin A nephropathy; DSA, donor specific antibody; XM, crossmatch; LDKT, living donor kidney transplantation; DDKT, deceased donor kidney transplantation; ATG, anti thymocyte globulin; BPAR, biopsy proven acute rejection; FK, tacrolimus; BKVAN, BK virus-associated nephropathy; OR, odd ratio; CI, confidence interval; ref, reference.

## Discussion

After KT, patients become immunocompromised and susceptible to BK viremia. Treatment of BKV replication involves reducing immunosuppression in patients with BK viremia before the progression to BKPVN, preventing irreversible allograft damage. BK viremia has been reported to have a positive predictive value of 30–50% for proven BKPVN during the window period 2–8 weeks prior^7^. The treatment guidelines were updated in 2019. Recipients undergo post-transplant screening monthly until month 9 and then every 3 months until 2 years post-transplantation. If the serum BKV DNA is detectable and exceeds 3 in the log10 scale, immediate treatment is recommended for recipients with stable renal function, while those with renal dysfunction should undergo a biopsy to confirm the presence of BKVAN. Immunosuppressant therapy involves a dose reduction of CNI by 25–50% in one or two steps, along with a 50% reduction in the antimetabolite drug, followed by discontinuation of the latter. However, there is currently no established treatment strategy for switching from tacrolimus to cyclosporine A (CsA) or mTOR inhibitor or from mycophenolic acid (MPA) to mTORi or leflunomide during reduction^7^. Our study aimed to compare the outcomes between two groups: one undergoing CNI reduction and MPA discontinuation in the triple regimen, and the other switching to mTORi during CNI discontinuation and MPA discontinuation.

We segmented patients based on the initial viral load and evaluated the outcomes of the two groups to facilitate practical comparison. Due to the retrospective nature of this study, there is heterogeneity in the initial viral load. As indicated in Table 1, there is a difference in initial viral load between the Reduction and Conversion groups (3.4 vs. 4.1, p < 0.001). We divided the initial viral load into intervals of 3–4 and 4–5 in the log10 scale and compared clinical outcomes. For the Conversion group, there were 31 individuals in the iVL 3–4 range and 26 individuals in the iVL 4–5 range. By evenly distributing the participants, we could observe the effects of initial viral load within the Conversion group. The reason for further dividing the iVL 4–5 group was due to the small number of patients with iVL greater than 5, and we believed that comparing treatment outcomes within similar ranges of initial viral load would be meaningful.

Our findings reveal several important insights into the management of BK viremia after KT. Firstly, in the iVL 3–4 range on the log10 scale, the Reduction group demonstrated comparable viral clearance and lower rates of BPAR compared to the Conversion group. These results suggest that a strategic reduction in immunosuppressive medications, particularly CNI and MPA agents, can effectively suppress BK virus replication without compromising allograft integrity. This aligns with findings from other studies, indicating that achieving viremia clearance through immunosuppression reduction alone is more readily attainable when BK viremia is in the range of 3–4 on the log10 scale, as opposed to cases where it exceeds 4 or when BKVAN is present^8–11^. Notably, the Reduction group exhibited a shorter time duration for viral clearance, potentially indicating a more rapid response to therapy. Moreover, the lower rates of BPAR in the Reduction group support the notion that judicious dose reduction of immunosuppressants can maintain graft stability while curbing viral replication. In addition, when the initial viral load is in the range of 3–4 on the log10 scale, differences in peak viral load are observed between the Reduction and Conversion groups (3.4 vs. 4.1, p < 0.001). Peak viral load reflects the highest viral load observed during treatment from the initial viral load. A higher peak viral load compared to the initial viral load reflects a slower response to treatment. The significantly higher peak viral load in the Conversion group indicates a poorer treatment response compared to the Reduction group.

On the other hand, in the iVL 4–5 range on the log 10 scale, although the Reduction group showed trends toward better viral clearance, the differences were not statistically significant. This suggested that while dose reduction of immunosuppressive agents may still be effective, it might have diminishing returns as viral load increases. It was worth noting that the risk of BPAR was higher in the Conversion group within this viral load range. This observation underscored the complexity of choosing appropriate treatment strategies, particularly for patients with higher viral loads.

Several studies have highlighted the potential of mTORi in addressing BKV replication, offering a unique perspective on BKVAN treatment strategies^12,13^. The rationale for incorporating mTOR inhibitor regimens stems from their interaction with the mTOR pathway and their influence on the differentiation of virus-specific CD8 memory T cells^14,15^. Previous research has underscored the capacity of mTOR inhibitors to exert a specific antiviral effect on BKV replication within tubular epithelial cells. Furthermore, in vitro investigations have shown that sirolimus, an mTOR inhibitor, exerts an inhibitory effect on BKV replication distinct from the activating impact of tacrolimus^16–18^. According to a meta-analysis, mTOR inhibitors have been reported to have a potential for use as a primary treatment or in the refractory or advanced stages of BKVAN, as they affect reducing the incidence of BK viremia and BKVAN^19^.

On the other hand, switching from CNI to sirolimus is supported by weak-level evidence in the 2019 guideline^7^. While there have been several studies comparing the benefits of mTORi-based immunosuppression to Tacrolimus-based regimens, there are limitations in the data regarding their effectiveness against BKVAN^20,21^. In our study, as in line with other research, we also could not confirm a positive effect. It was challenging to elucidate the effect of BKVAN due to the inability to perform biopsies at the same time point for all patients. Furthermore, reports indicate that converting to mTOR inhibitor can increase the rate of acute rejection^22^. In our study, we followed up on BPAR within 1 year after diagnosing and treating BK viremia. The results indicated a higher incidence of BPAR in the Conversion group among patients with an iVL level of 3–4 on the log 10 scale.

We defined persistent viremia as the persistence of detectable viremia after the 3 months of treatment for these reasons. First, the median duration for clearance of viremia in the entire patient cohort was 3.0 months in this study. Notably, a similar investigation was reported, where the definition of persistent viremia was adopted to compare the efficacy of antiviral and combined IVIG therapies versus conventional therapy after 3 months of treatment for BK virus nephropathy^23^. Based on this definition, our patients were also evaluated for risk factors associated with persistent viremia, and it was observed that a higher peak viral load was significantly associated with an increased risk (Hazard ratio [HR] 4.49, 95% CI 2.60–7.75, p < 0.001).

There are several limitations to this retrospective cohort study. First, the inherent biases associated with retrospective studies, such as selection bias and information bias, may affect the results. The selection of induction agents was also not pre-controlled. However, immunological risk was not different between groups and the induction agent was not a factor influencing the outcome. Second, the sample size and follow-up periods may be limited, and the study may not have enough power to detect small differences between treatment groups. Third, as this study was conducted in a two-center setting, the results may not be generalizable to other populations or settings. Lastly, kidney biopsy protocols were different between the two participating centers and the effect of treatment strategies could not be compared in patients with biopsy-proven BKVN.

This retrospective cohort study aimed to evaluate and compare the outcomes of two treatment methods for BKV-associated diseases in kidney transplant recipients. The present study did not support switching from tacrolimus to mTORi could serve as an alternative treatment for BK viremia treatment in kidney transplant recipients. Overall, this study contributed to the existing literature and may have implications for the management of BK viremia after KT. Further research in larger multicenter cohorts is warranted to confirm our findings and establish optimal treatment strategies for BKV-associated diseases.

## Methods

### Study design and population

This retrospective cohort study was conducted on patients aged 18 years and older who underwent living or deceased donor KT from January 1, 2016, to June 30, 2020, at two tertiary hospitals in Seoul, Korea: Seoul National University Hospital (SNUH) and Samsung Medical Center (SMC). Patients who received simultaneous transplantation of kidneys and other organs, those with loss to follow-up and those who received alternative treatment modalities such as leflunomide, cidofovir, cyclosporine A (CsA), or everolimus were excluded from the study.

### Screening protocols for BK viremia

In SNUH, serum BK viral load was assessed using the quantitative polymerase chain reaction (qPCR) method at specific intervals: postoperative day 10, 1 month, and bi-monthly up to the first year post-kidney transplantation. Subsequently, assessments were conducted every 6 months. In cases where the virus was detected, the serum BK viral load was monitored every month. In SMC, urine BK virus DNA was checked at postoperative 1, 5, 9, 16, 24, 36, and 48 weeks. If BK virus DNA was detected in urine, the qPCR analysis of the urine BKV DNA viral load was performed. If the urine BKV DNA viral load was 7 or above 7 on log10 scales, the serum BK viral load was assessed. If the serum BK viral load was 2 or above 2 on log-10 scales, the subsequent measurement was taken on a monthly or bi-monthly basis. The clearance of the serum BK viral load was defined as a reduction in viral load below the detection threshold (< 100 copies/ml).

The protocol biopsy was conducted in SNUH at 1 year and 5 years post-transplantation, while it was performed at 1 year post-transplantation in SMC. In SNUH, the indication biopsy was only performed for patients with BK viremia when concurrent renal dysfunction was present. On the other hand, in SMC, the indication biopsy was conducted for all BK viremia patients unless there were contraindications for the kidney biopsy such as coagulopathy.

The treatment for BPAR involved intravenous methylprednisolone at a dose of 300 to 500 mg daily for 3 to 5 days for T cell-mediated rejection. Also, antibody-mediated rejection is treated with plasmapheresis, rituximab at a single dose of either 200 mg or 375 mg/m^2^ and intravenous immunoglobulin at a dose 100 mg/kg.

### Treatment groups

Immunosuppression at post KT were triple regimen: mycophenolate acid (1 g/day), tacrolimus (with trough level targeted between 8 and 10 ng/ml before 3 months, 7 and 8 ng/ml before 6 months, 6 and 7 before 1 year, and 5 and 6 ng/ml after 1 year post KT), and prednisolone (given at 500 mg pre-operatively, then progressively tapered to 5 mg/day).

According to the treatment algorithm outlined by Hirsch et al., BKPVN is considered probable when measured at 1000 copies/mL or above^7^. This recommendation is based on the assertion that BKPVN is likely to occur in kidney transplant patients with sustained plasma BK viremia exceeding 1000 copies/mL. Therefore, patients with the serum BK viral load measurement of 1000 copies/ml or higher were included in this study and classified into two groups based on their treatment methods. In the Reduction group, the dose of tacrolimus was reduced by 25–50%, and the target tacrolimus trough level was set at < 6 ng/ml. Additionally, the dose of the antimetabolite drug was reduced by 50% or discontinued completely. In the Conversion group, patients discontinued the use of tacrolimus, reduced or discontinued antimetabolites, and added the sirolimus at a median level of 8 ng/ml.

### Data collection

Demographic, clinical, and laboratory data of the enrolled patients were collected from electronic medical records. Demographic information included age, sex, underlying diseases, comorbidities, and other transplantation-related factors. Clinical information included immunosuppressive regimens and antiviral treatments. Laboratory data included serum creatinine, estimated glomerular filtration rate (eGFR), BK viral load (copies/mL).

### Study endpoints

The efficacy and the safety outcomes of two treatments were evaluated. For the efficacy evaluation, persistent viremia after 3 months was defined as the continued presence of BK viremia on consecutive measurements at 3 months after the initial diagnosis. To measure the safety outcomes, the BPAR including borderline rejection within the first year after the initiation of BKV treatment. Secondary endpoints include clinical outcomes such as serum creatinine and eGFR levels, the time duration required for viral load clearance, the velocity of viral clearance, the occurrence of BKVAN, the graft survival, and the patient survival. Setting the criterion for viral clearance at less than 100 copies/mL was due to the hospital's reporting system of viral load PCR tests with 100 copies/mL as the lower limit. The time duration for clearance was defined as the interval from the initial detection of BK viral load until it decreased below the detection threshold (< 100 copies/mL). The velocity of viral clearance (copies/mL/month) is calculated by dividing the peak viral load (copies/mL) by the time duration required for viral load clearance (months).

### Statistical analysis

IBM SPSS Statistics for Windows ver. 22.0 (IBM Corp. Armonk, NY, USA) was used for the statistical analyses. Descriptive statistics were used to summarize the demographic, clinical, and laboratory data of the study population. Continuous variables were reported as mean ± standard deviation or median with interquartile range, depending on the distribution of the data. Categorical variables were presented as proportions. Comparisons between different treatment methods were performed using appropriate statistical tests, such as the chi-squared test or Fisher's exact test for categorical variables, and the t-test or Mann–Whitney U test for continuous variables. Multivariable logistic regression analysis was performed to identify factors associated with persistent viremia and other clinical outcomes. Statistical significance will be set at p < 0.05.

### Ethical considerations

This study has been approved by the Institutional Review Boards of SNUH and SMC was conducted in accordance with the principles of the Declaration of Helsinki. As this was a retrospective study, informed consent from the patients was waived (SNUH IRB No 2205–022-1320 and SMC IRB No 2023–10-106). This manuscript attests that no organs or tissues were procured from prisoners.

## Data Availability

The datasets generated during and/or analyzed during the current study are available from the corresponding author on reasonable requests.
